# Chalcone Derivatives Enhance ATP-Binding Cassette Transporters A1 in Human THP-1 Macrophages

**DOI:** 10.3390/molecules23071620

**Published:** 2018-07-03

**Authors:** I-Jou Teng, Min-Chien Tsai, Shao-Fu Shih, Bi-Feng Tsuei, Hsin Chang, Yi-Ping Chuang, Chin-Sheng Lin, Ching-Yuh Chern, Sy-Jou Chen

**Affiliations:** 1 Graduate Institute of Medical Sciences, National Defense Medical Center, Taipei 11490, Taiwan; topy900703@gmail.com; 2 Department of Physiology and Biophysics, Graduate Institute of Physiology, National Defense Medical Center, Taipei 11490, Taiwan; mctsaisyy@gmail.com; 3 Division of Cardiology, Department of Medicine, Tri-Service General Hospital, National Defense Medical Center, Taipei 11490, Taiwan; evarei080@gmail.com (S.-F.S.); littlelincs@gmail.com (C.-S.L.); 4 Graduate Institute of Life Sciences, National Defense Medical Center, Taipei 11490, Taiwan; befun1214@gmail.com; 5 Department of Applied Chemistry, National Chiayi University, Chiayi City 60004, Taiwan; s1060317@mail.ncyu.edu.tw (H.C.); cychern@mail.ncyu.edu.tw (C.-Y.C.); 6 Department and Graduate Institute of Microbiology and Immunology, National Defense Medical Center, Taipei 11490, Taiwan; ypchuang@mail.ndmctsgh.edu.tw; 7 Department of Emergency Medicine, Tri-Service General Hospital, National Defense Medical Center, Taipei 11490, Taiwan; syjou.chen@gmail.com

**Keywords:** atherosclerosis, cholesterol efflux, ATP-binding cassette transporters A1, chalcone derivative

## Abstract

Atherosclerosis is a process of imbalanced lipid metabolism in the vascular walls. The underlying pathology mainly involves the deposition of oxidized lipids in the endothelium and the accumulation of cholesterol in macrophages. Macrophages export excessive cholesterol (cholesterol efflux) through ATP-binding cassette transporter A1 (ABCA1) to counter the progression of atherosclerosis. We synthesized novel chalcone derivatives and assessed their effects and the underlying mechanisms on ABCA1 expression in macrophages. Human THP-1 macrophages were treated with synthetic chalcone derivatives for 24 h. In Western blot and flow cytometry analyses, a chalcone derivative, (*E*)-*1-(3,4-diisopropoxyphenyl)-3-(4-isopropoxy-3-methoxyphenyl)prop- 2-en-1-one* (**1m**), was observed to significantly enhance ABCA1 protein expression in THP-1 cells (10 µM, 24 h). Levels of mRNA of ABCA1 and liver X receptor alpha (LXRα) were quantified using a real-time quantitative polymerase chain reaction technique and were found to be significantly increased after treatment with the novel chalcone derivative **1m**. Several microRNAs, including miR155, miR758, miR10b, miR145, miR33, and miR106b, which functionally inhibit ABCA1 expression were suppressed after treatment with 1m. Collectively, 1m increases ABCA1 expression in human THP-1 macrophages. The mechanisms involve the activation of the LXRα-ABCA1 pathway and suppression of certain microRNAs that regulate ABCA1 expression.

## 1. Introduction

Atherosclerosis is the leading cause of cardiovascular diseases including myocardial infarction, stroke, unstable angina, and sudden cardiac death worldwide [1]. The underlying pathology has been considered a chronic proinflammatory consequence of excess deposition of oxidized low-density lipoprotein (oxLDL) in arterial walls [2,3]. The formation of foam cells, namely lipid laden macrophages, in the vascular intima plays a critical role in the initiation and progression of endothelial dysfunction in atherosclerosis [4,5,6].

Reverse cholesterol transport, a process by which cholesterol is transferred from peripheral tissues and cells to the liver for biliary secretion, contributes to protection from atherosclerosis. Excess oxLDL is considered to be counterbalanced by cholesterol efflux mediated mainly by reverse cholesterol transporters (RCT) [7]. ATP-binding cassette transporter A1 (ABCA1), a major RCT, exports excess cholesterol from cells to apolipoprotein A-I and mediates the synthesis of high-density lipoprotein (HDL) cholesterol. Human epidemiology studies have shown an inverse relationship between HDL cholesterol and cardiovascular events [8,9,10]. Moreover, cholesterol efflux capacity is inversely associated with the incidence of cardiovascular events demonstrated in a population-based cohort [11].

Chalcone (1,3-diphenyl-2-propen-1-one) is a phenolic compound that is widely biosynthesized in plants [12]. Epidemiological studies have suggested that increased intake of polyphenols from fruits and vegetables reduces the risk of cardiovascular disease. Naturally occurring chalcones as well as synthetic chalcone analogues have indicated numerous pharmaceutical effects, including anti-inflammatory, antioxidant, antiapoptosis, antiparasite, and antitumor activities [13,14,15,16,17,18,19]. Because of privileged structural and chemical properties, chalcones are excellent at modifying new drug designs and development [12]. However, the antiatherosclerotic effects of chalcones remain poorly investigated [20]. A study on 2-hydroxy-4′-methoxychalcone, a synthetic chalcone derivative, reported that this derivative significantly inhibited the oxLDL-induced proliferation of human aortic smooth muscle cells [21]. Synthetic indole–chalcone fibrates exhibited in vitro antioxidant and in vivo antidyslipidemic effects in mice [22]. Although few studies have started to focus on its potential in atherosclerosis prevention, no studies have investigated its effects on regulating ABCA1.

Therefore, the present study investigated the effects of chalcone on the expression of ABCA1 in macrophages. We synthesized several chalcone derivatives and identified a novel chalcone derivative that significantly increased the expression of ABCA1. The mechanism works by enhancing the liver X receptor alpha (LXRα) signaling pathway and inhibiting several microRNAs (miRNA) that regulate the expression of ABCA1.

## 2. Results

### 2.1. Chemistry

**Figure 1:**
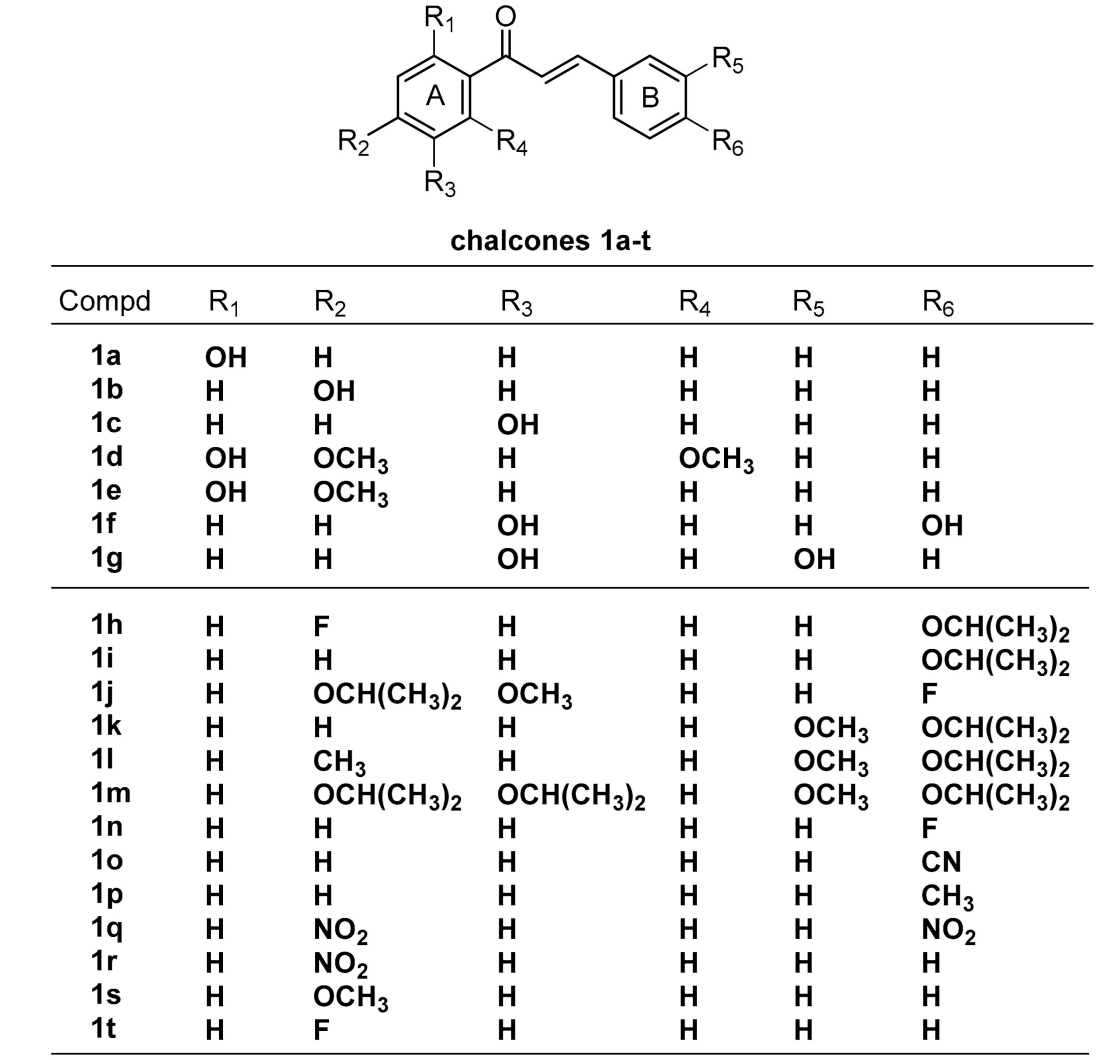
List of chalcone derivatives **1a**–**t**.

**Scheme 1:**
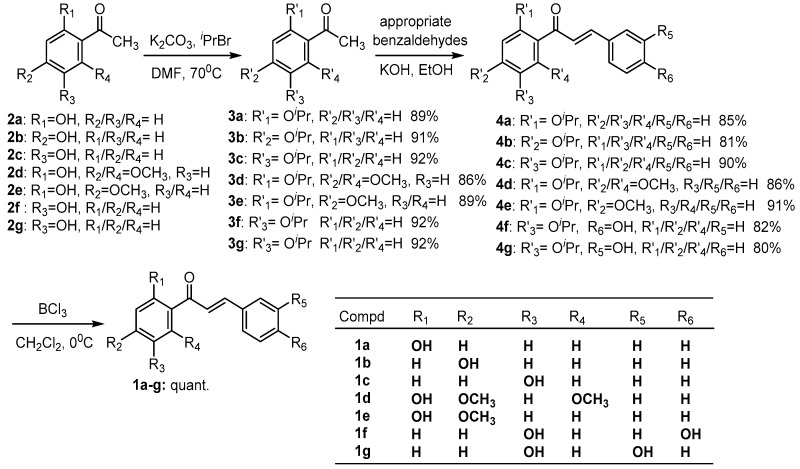
Chalcone derivatives **1a**–**g**.

For this study, we synthesized 20 chalcones **1a**–**t** (Figure 1). These Aldol intermediates were obtained using a procedure similar to the one described previously in References [23,24,25,26,27]. Intramolecular hydrogen bonding such as that observed in **1a**–**g** is known to prevent Aldol reactions. Therefore, *O*-isoproxyl-acetophenones **3a**–**g** were used as the starting materials for our synthesis. The preparation of *O*-isoproxyl- acetophenones **3a**–**g** was straightforward. Acetophenone **2a**–**g** were protected with isopropyl bromide and potassium carbonate in dimethylformamide at around 95% yield. The isolated products **3a**–**g** were then reacted with appropriate benzaldehydes with 5N KOH to offer the intermediates **4a**–**g**. The *O*-isopropyl ether was removed quantitatively with BCl_3_ to afford the target chalcones **1a**–**g** (Scheme 1), and each compound displayed > 95% purity based on the proton nuclear magnetic resonance (^1^H-NMR) results [23]. 

**Scheme 2:**
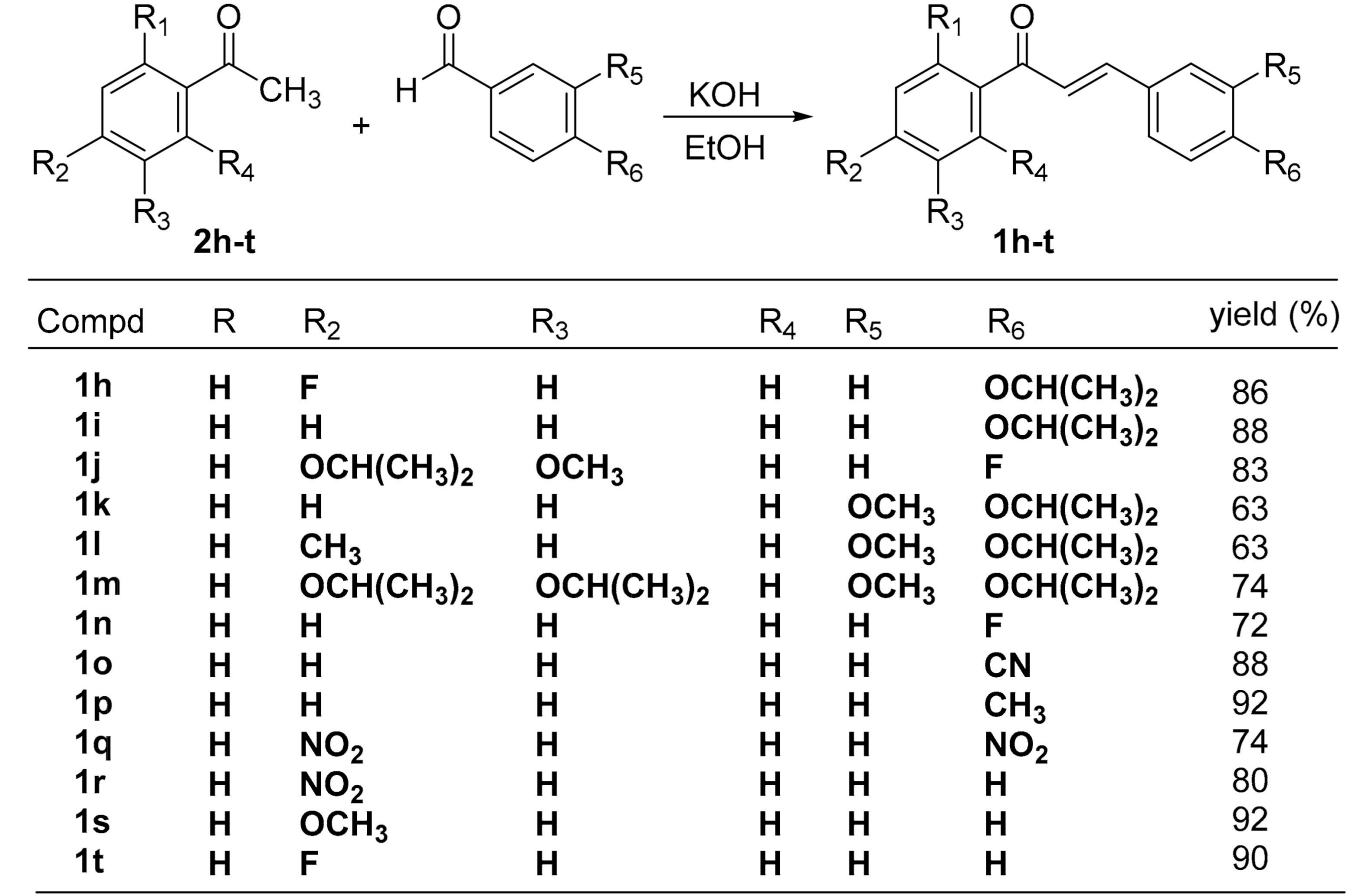
Chalcone derivatives **1h**–**t.**

The starting materials **2h**–**m** used in this study are new and have not been reported before. We were the first group to synthesize the target compounds **1h**–**m**. In the present study, we employed the same methodology to synthesize the new compounds **1h**–**m** and the known compounds **1o**–**t** (Scheme 2). 

### 2.2. Screening for ABCA1 Expression in Human THP-1 Macrophages Treated with Chalcone Derivatives

**Figure 2:**
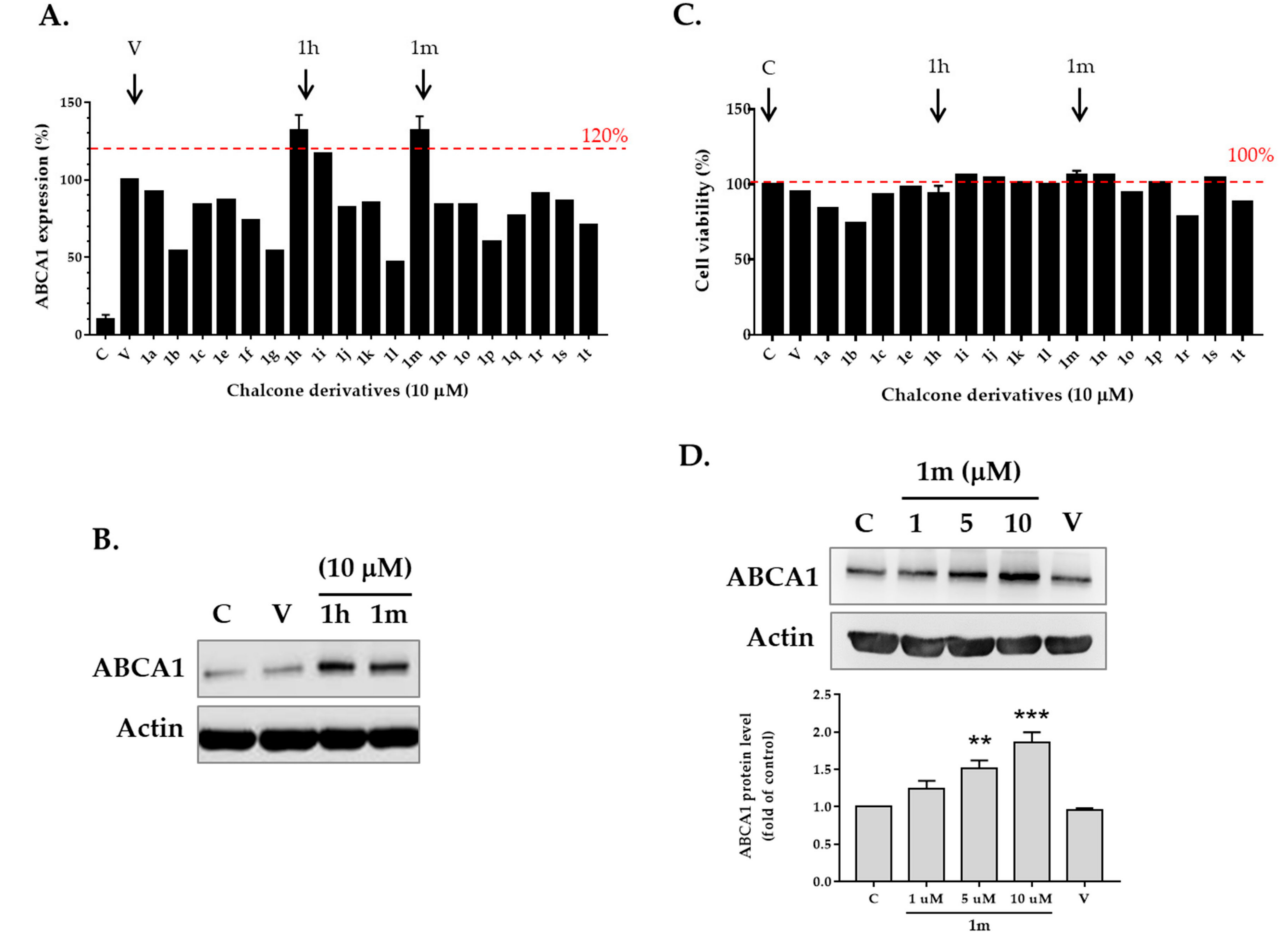
Effects of chalcone derivatives on the expression of ABCA1 in human THP-1 macrophages. (**A**) Human THP-1 macrophages were treated with control (**C**), vehicle (V; 0.1% DMSO) and various chalcone derivatives at a concentration of 10 µM for 24 h; cells were then washed and stained with ABCA1 antibody. The expression of cell surface ABCA1 was analyzed using flow cytometry. (**B**) Cell lysates were collected after treatment with the selected chalcones **1h** and **1m** at 10 µM for 24 h. The expression of ABCA1 and Actin in THP-1 macrophages was determined by Western blot. (**C**) THP-1 macrophages were incubated with vehicle (0.1% DMSO) or chalcone compounds (10 μM) for 24 h. Cells were then incubated with MTT for six hours. Cell viability was measured by the amount of dissolved reduced MTT crystals using an enzyme-linked immunosorbent assay reader; **1m** was nontoxic to THP-1 macrophages and was selected for subsequent studies. (**D**) THP-1 macrophages were treated with the indicated concentrations of **1m**. The expression of ABCA1 protein was determined by Western blot. Data are shown by representative bands and presented as the mean ± SEM of three independent experiments in triplicate after densitometric analysis. (** *p* < 0.01, *** *p* < 0.001 vs. control).

Excessive cholesterol accumulation in macrophages aggravates foam cell formation. ABCA1 is one of the most crucial RCTs facilitating the removal of intracellular cholesterol in macrophages. Subsequently, flow cytometry was used to screen chalcon derivatives that potentially enhance ABCA1 expression in human THP-1 macrophages. THP-1 macrophages were treated with synthetic chalcone derived compounds at 10 μM for 24 h. As shown in Figure 2A, ABCA1 expression significantly increased in the THP-1 macrophages treated with **1h** and **1m**. The results were further confirmed through Western blot analysis, which showed enhanced ABCA1 protein expression in the THP-1 macrophages treated with **1h** and **1m** compared with the vehicle (Figure 2B). To determine whether these chalcone derivatives affect cell viability, cell cytotocixity was analyzed using an MTT (3-(4,5-Dimethylthiazol-2-yl)-2,5-diphenyl-tetrazolium bromide) assay in the presence of various chalcone compounds at 10 μM for 24 h. The results of the MTT assay showed that most chalcone analogues significantly suppressed cell viability, except for **1m** (Figure 2C). Therefore, **1m** was selected for subsequent studies. These results indicated that 1m is not cytotoxic to human THP-1 cells and that 1m increases ABCA1 expression without inducing cell death. Furthermore, to determine the different dose effects on ABCA1 expression, THP-1 cells were treated with indicated concentrations (1, 5, and 10 μM) of **1m** for 24 h. The results showed that 1m significantly and concentration-dependently increased the expression of ABCA1 (Figure 2D).

### 2.3. Effects of Chalcone ***1m*** on the Expression of Transcription Factors LXRα and PPARγ

**Figure 3:**
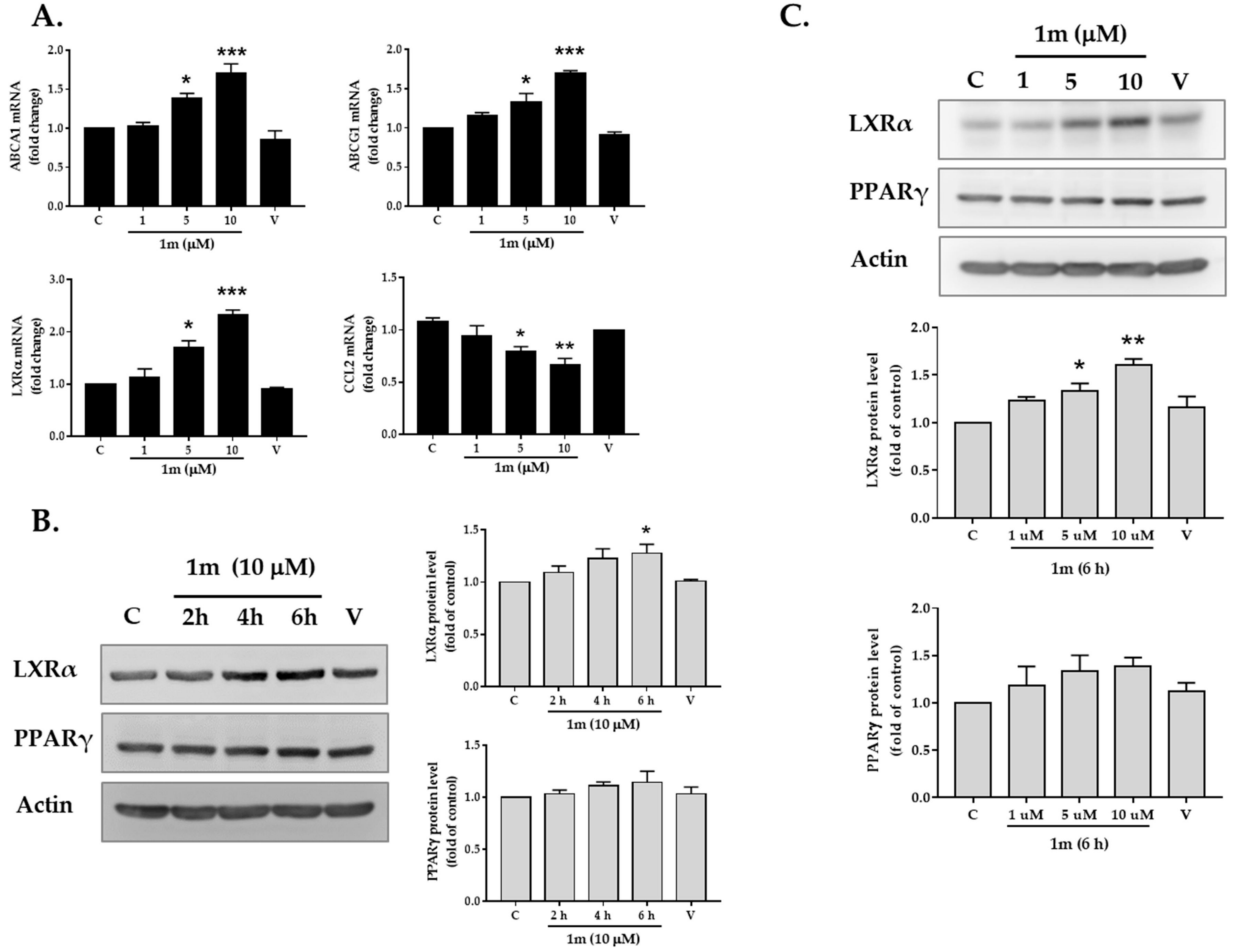
Protein and mRNA expression of transcriptional factor LXRα was enhanced in THP-1 macrophages treated with chalcone **1m**. (**A**) THP-1 macrophages were treated with indicated concentrations (1, 5, and 10 μM) of 1m for 24 h. Expression of mRNA of LXRα, ABCA1, ABCG1, and CCL2 was determined using qRT-PCR analysis. The relative expressions of mRNA are shown. (**B**) THP-1 macrophages were treated with 10 μM of 1m for the time indicated (2, 4, and 6 h), or (**C**) treated with indicated doses (1, 5, and 10 μM) for 6 h. Cell lysates were collected and protein expression of LXRα, PPARγ, and Actin were determined by Western blot. Data are shown by representative bands and presented as the mean ± SEM of three independent experiments in triplicate after densitometric analysis. (* *p* < 0.05, ** *p* < 0.01, *** *p* < 0.001 vs. control).

Liver X receptors (LXRs) are sterol-activated transcriptional factors. LXRα targets promoter genes that regulate RCTs, and it is critical for the regulation of peroxisome proliferator-activated receptor gamma expression (PPARγ) and several downstream genes such as chemokine (C–C motif) ligand 2 (CCL2). To investigate whether LXRα is involved in **1m**-induced ABCA1 expression at the transcriptional level, several mRNAs were assessed. As shown in Figure 3A, treatment with **1m** for 24 h increased levels of mRNA of LXRα, ABCA1, and ATP-binding cassette transporter G1(ABCG1) and decreased CCL2 mRNA expression in THP-1 macrophages in a concentration-dependent manner (5 and 10 μM). Subsequently, we investigated the protein expression of LXRα and PPARγ. THP-1 macrophages were incubated with 10 μM of **1m** for 2, 4, and 6 h. The protein expression of LXRα significantly increased at 6 h, but that of PPARγ did not (Figure 3B). Treatment of **1m** also concentration-dependently enhanced LXRα protein expression at 6 h, but it did not alter the expression of PPARγ (Figure 3C). These results suggest that LXRα is involved in **1m**-induced ABCA1 expression in THP-1 macrophages.

### 2.4. Effects of Chalcone **1m** on the Expression of MicroRNAs that Regulate ABCA1

**Figure 4:**
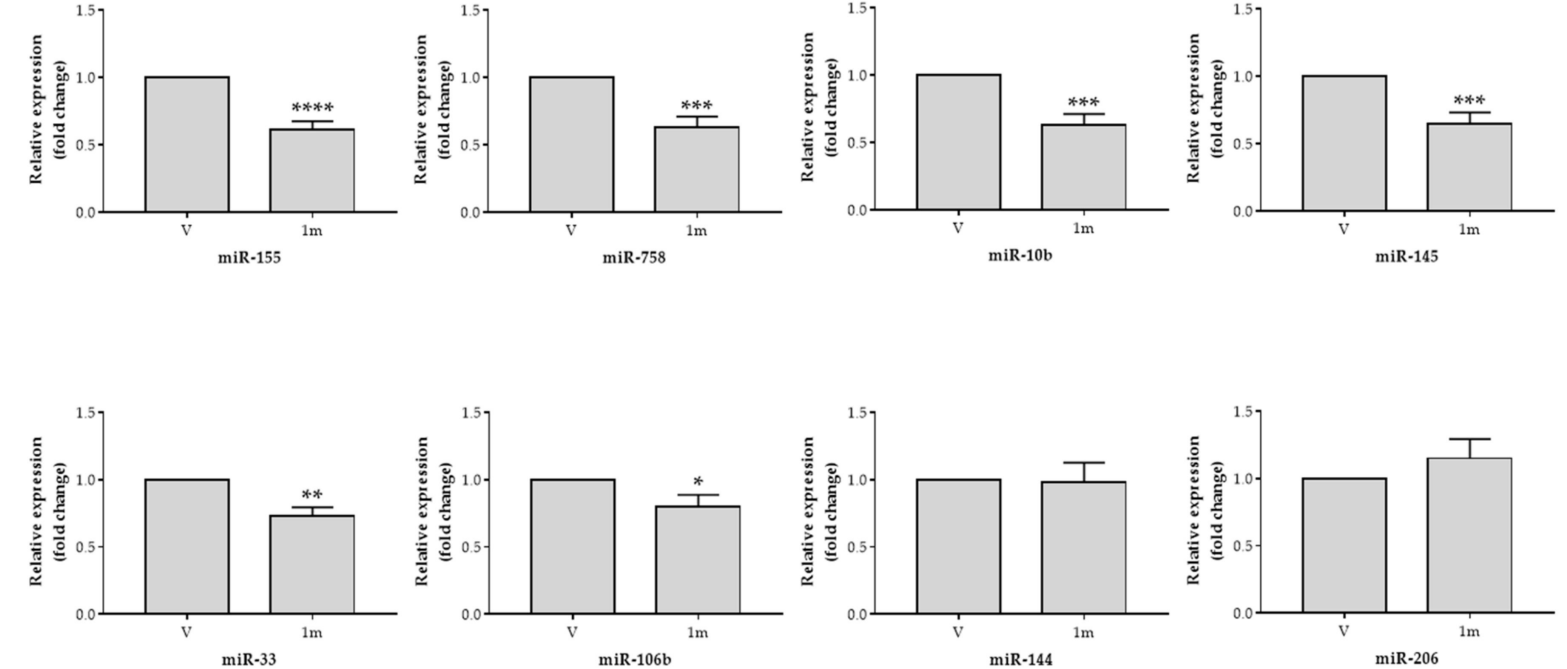
MicroRNAs were suppressed in THP-1 macrophages treated with chalcone **1m**. THP-1 macrophages were incubated with a vehicle control (V) and 10 μM of **1m** for 24 h and were lysed for measuring the indicated microRNAs. The levels of specific microRNA (miR144, miR33, miR106b, miR155, miR758, miR145, miR10b, and miR206) were measured using qRT-PCR. Data are shown by the relative expressions of miRNA. (* *p* < 0.05, ** *p* < 0.01, *** *p* < 0.001, **** *p* < 0.0001 vs. vehicle control).

A pool of miRNAs is known to target several genes that are critical for the regulation of cholesterol metabolism. Specific candidates such as miR10b, miR33, miR106b, miR144, miR145, miR155, miR206, and miR758, which have been demonstrated to directly and indirectly inhibit the expression or function of ABCA1, were selected [28,29,30,31,32,33,34,35]. The results of real-time quantitative polymerase chain reaction (qRT-PCR) (Figure 4) revealed that treatment with **1m** for 24 h reduced the expression of miR155, miR758, miR10b, miR145, miR33, and miR106b, but did not affect the expression of miR144 and miR206. 

## 3. Discussion

**Figure 5:**
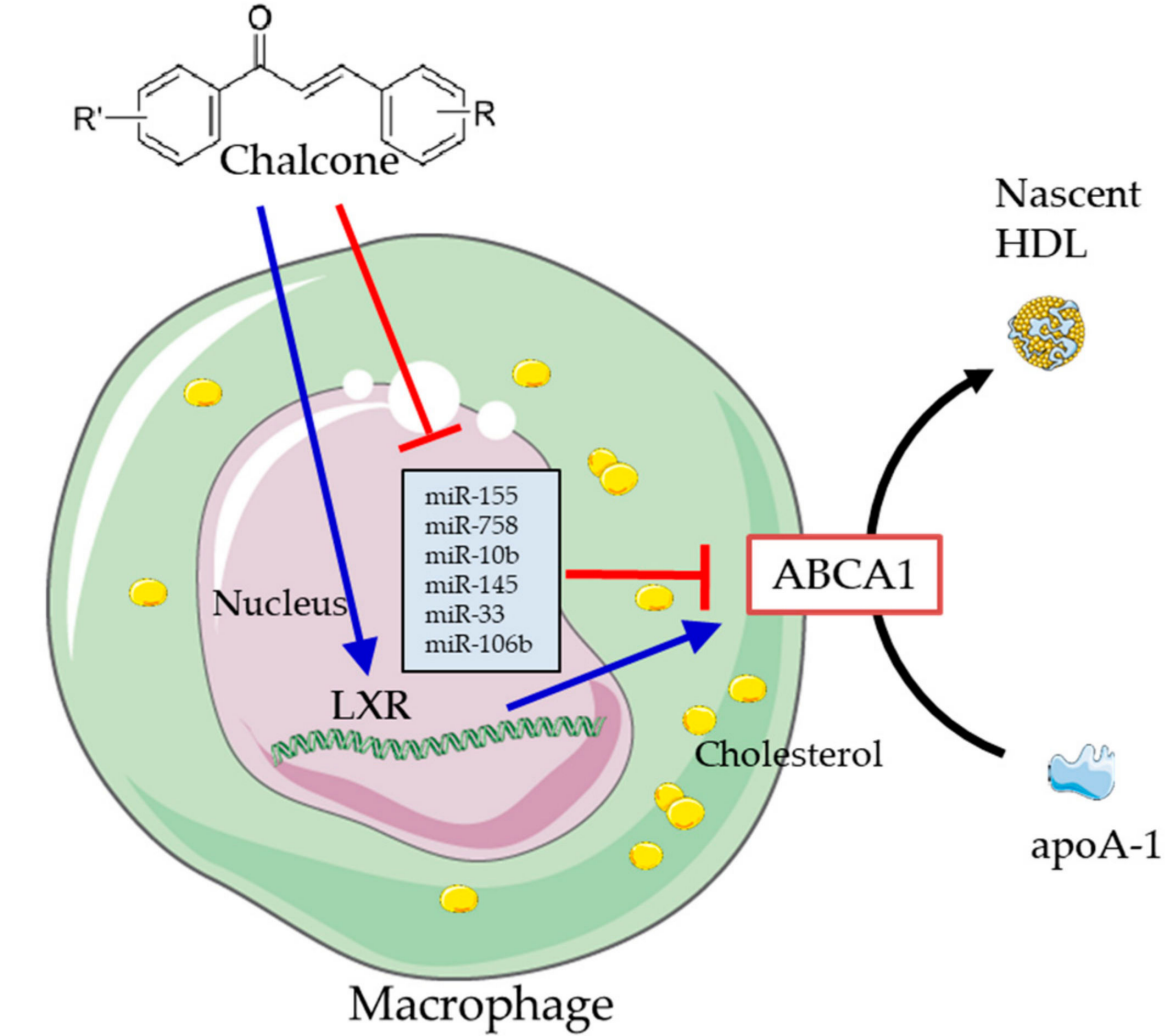
Potential mechanisms of the chalcone derivative (**1m**) in regulation of ABCA1 expression in macrophage.

Chalcone-based derivatives exert various biological functions beneficial to the cardiovascular system. However, the antiatherosclerotic effects have been rarely evaluated until recently. We synthesized several novel chalcone derivatives and determined that **1m** can significantly enhance ABCA1 expression in THP-1 macrophages; **1m** significantly increased ABCA1 protein and mRNA expression in THP-1 macrophages. The mechanisms may be partly mediated by the LXRα-ABCA1 pathway and the inhibition of several microRNAs that regulate ABCA1 expression (Figure 5). This novel synthetic chalcone derivative that targets ABCA1 critical in foam cell formation can be beneficial to the prevention of atherosclerosis.

For a newly developed drug or chemical, it is generally considered that less than 10 μM (1–10) concentration of a drug in plasma is accepted because higher than 10 μM of a drug might inhibit necessary enzymes [36]. In the study by Peluso MR [37], Xanthohumol (chalcone) at 20 μM was used to test its anti-inflammatory effects on THP-1 cells, suggesting that 20 μM of Xanthohumol exerts no cytotoxicity effect on THP-1 cells. Moreover, in the study of in vitro structure-toxicity relationship of chalcones in human hepatic stellate cells, nineteen chalcones were analyzed for their structure related toxicity. Seventeen out of 19 studied chalcones showed an IC_50_ > 50 μM by the MTT assay, and nearly all (18/19) studied chalcones showed decreased cell viability at 50 μM [38]. Accordingly, we chose the concentration of 10 μM as a reasonable initial screening concentration in the current study.

Peroxisome proliferator-activated receptors (PPARs) are nuclear receptors that control transcriptional signals involved in processes of lipid metabolism, including fatty acid oxidation, fat cell development, and lipoprotein metabolism [39]. PPARγ activation can decrease the rate of cholesterol esterification by reducing acetyl-coenzyme acetyltransferase 1 mRNA levels, and controls cholesterol efflux in human macrophages. Furthermore, the activation of PPARγ reflects the nature of its anti-inflammatory properties. Liu et al. [21] studied a synthetic 2-hydroxy-4′-methoxychalcone in human aortic smooth muscle cells and demonstrated that it significantly inhibited the proliferation of oxLDL–induced aortic smooth muscle cells. The effects manifest through the activation of PPARγ, inhibition of p44/42 mitogen-activated protein kinase, and the progression of the cell cycle. However, **1m** did not alter the expression of PPARγ, but enhanced the expression of LXRα in THP-1 macrophages in the present study. Previous studies have shown that PPARs cooperate with LXRs to enhance transcription of the ABCA1 gene and that the activation is in an LXRα-dependent manner, suggesting that LXRα play a crucial role in the regulation of ABCA1 [40]. In this study, we demonstrated that treatment with **1m** in THP-1 macrophages significantly increase LXRα but not PPARγ expression, indicating that LXRα instead of PPARγ is the main target of this pharmacological action. The results are also in consistence with some reports, suggesting that the activation of LXRα and ABCA1 expression does not need pre-activation of PPARγ [41,42]

Liver X receptors (LXRs) are nuclear receptors, which have pivotal roles in regulating cholesterol homeostasis [43]. These receptors are ligand-dependent and control the expression of genes involved in the uptake, transport, efflux, and excretion of cholesterol in a tissue-dependent manner [44]. Treatment with LXRα activator increases expression of ABCA1 mRNA and promotes cholesterol efflux to apoA-I in macrophages [45]. LXRs have been identified as inflammatory regulators that suppress genes involved in inflammation, such as the C–C motif ligand 2 (CCL2) gene; furthermore, they can exert antiatherosclerotic effects independent of cholesterol efflux pathways [46,47,48]. C–C motif ligand 2 is a member of the CC chemokine family and is capable of suppressing HDL cholesterol internalization and cholesterol efflux [49,50]. Our results show that 1m selectively upregulates LXR and its downstream suppression gene, but not PPARγ, which suggests that 1m might specifically upregulate ABCA1 at the transcriptional level through the LXR-ABCA1 pathway.

MicroRNAs (miRNAs) are short, noncoding RNAs (18–25 nucleotides) that are incorporated into the RNA-induced silencing complex where it binds to the 3′-untranslated region of its target genes, promoting translational repression and mRNA degradation [51]. Studies have shown that ABCA1 expression is tightly regulated at the posttranscriptional level by miRNAs, including HDL biogenesis, cholesterol efflux, cholesterol uptake in the liver, and bile acid synthesis and secretion [52]. Numerous miRNAs have been shown to regulate ABCA1 expression. For example, antagonists of miR-33 increase hepatic ABCA1 expression and elevated plasma HDL cholesterol levels [28]. Previous studies suggested that several microRNAs, including miR-145, miR-106b, miR-206, miR-155, miR-144, miR-758, miR-10b, and miR-33 are involved in manipulation of ABCA1 expression [28,29,30,31,32,33,34,35]. We found that treatment with 1m reduced the expression of miR-155, miR-758, miR-10b, miR-145, miR-33, and miR-106b, but did not affect the expression of miR-144 and miR-206, which suggests that **1m** may participate in regulation of ABCA1 expression at a posttranscriptional level. 

Various biological activities of chalcone analogues have been identified. Specific characteristics that are beneficial for protecting against atherosclerosis include anti-inflammatory, antioxidizing, and antiplatelet properties. The present study proposed a novel synthetic chalcone compound and demonstrated its superior effects on increasing the expression of ABCA1, a critical cholesterol regulator in macrophages. Although we found that treatment with 1m significantly increased ABCA1 expression, the effect of 1m on cholesterol efflux has not been determined, a limitation in the present study. Further in vitro and in vivo cholesterol efflux studies are warranted to investigate its effect on atherosclerotic cardiovascular disease.

## 4. Materials and Methods 

### 4.1. Synthesis of Chalcone Analogues

Proton (300 MHz) and carbon (75 MHz) NMR spectra were recorded on a Varian Mercury-300 NMR spectrometer (Agilent, Santa Clara, CA, USA). Chemical shifts were reported on the δ scale as parts per million (ppm) downfield from tetramethylsilane (TMS), which was used as an internal reference. Mass spectra were measured using a VG Analytical Model 70–250 s Mass Spectrometer (Varian, Palo Alto, CA, USA). All reagents were used as obtained commercially.

(*E*)*-1-(2-hydroxy-phenyl)-3-phenyl-propenone* (**1a**) [24]. ^1^H-NMR (CDCl_3_): δ 12.80 (1H, s, –OH), 7.93 (1H, d, *J* = 15.6 Hz), 7.93 (1H, d, *J* = 8.0 Hz), 7.66 (1H, d, *J* = 15.6 Hz), 7.68–7.65 (1H, m), 7.55–7.42 (5H, m), 7.03 (1H, dd, *J* = 8.0 Hz, 1.0 Hz), 6.95 (1H, td, *J* = 7.2 Hz, 1.0 Hz); ^13^C-NMR (CDCl_3_): δ 193.8, 163.5, 145.4, 136.4, 134.5, 130.9, 129.6, 129.0, 128.6, 120.0, 119.9, 118.8, 118.6.

(*E*)*-1-(4-hydroxy-phenyl)-3-phenyl-propenone* (**1b**) [24]. ^1^H-NMR (CDCl_3_): δ 8.00 (2H, d, *J* = 8.8 Hz), 7.80 (1H, d, *J* = 15.6 Hz), 7.63 (2H, dd, *J* = 2.6 Hz, 2.0 Hz), 7.53 (1H, d, *J* = 15.6 Hz), 7.42–7.40 (3H, m), 6.93 (2H, d, *J* = 8.8 Hz), 2.10–1.90 (1H, br s, –OH); ^13^C-NMR (CDCl_3_): δ 188.4, 162.9, 143.8, 136.4, 132.0, 131.2, 131.0, 129.8, 129.4, 123.0, 116.2.

(*E*)*-1-(3-hydroxy-phenyl)-3-phenyl-propenone* (**1c**) [24]. ^1^H-NMR (CDCl_3_): δ 7.83 (1H, d, *J* = 15.6 Hz), 7.66–7.58 (3H, m), 7.51 (1H, d, *J* = 15.6 Hz), 7.45–7.34 (5H, m), 7.14 (1H, ddd, *J* = 8.0 Hz, 2.4 Hz, 1.2 Hz), 4.8–4.2 (1H, br s, –OH); ^13^C-NMR (CDCl_3_): δ 190.9, 156.5, 145.5, 139.4, 134.7, 130.7, 129.9, 129.0, 128.6, 121.9, 120.9, 120.5, 115.2.

(*E*)*-1-(2,6-Dihydroxy-4-methoxy-phenyl)-3-phenyl-propenone* (**1d**) [25]. ^1^H-NMR (d_6_-acetone): δ 12.07 (2H, br s, –OH), 8.25 (1H, d, *J* = 15.6 Hz), 7.73 (1H, d, *J* = 15.6 Hz), 7.71–7.66 (2H, m), 7.46–7.40 (3H, m), 6.04 (2H, s), 3.81 (3H, s); ^13^C-NMR (d_6_-acetone): δ 193.4, 167.2, 165.4, 142.9, 136.4, 130.9, 129.8, 129.1, 128.3, 106.2, 94.6, 55.8.

(*E*)*-1-(2-hydroxy-4-methoxy-phenyl)-3-phenyl-propenone* (**1e**) [53]. ^1^H-NMR (CDCl_3_): δ 13.48 (1H, br s, –OH), 8.10 (1H, d, *J* = 15.6 Hz), 6.45–7.94 (8H, m), 6.41 (1H, d, *J* = 15.6 Hz), 3.86 (3H, s). 

(*E*)*-1-(3-hydroxyphenyl)-3-(4-hydroxyphenyl)prop-2-en-1-one* (**1f**) [54]. ^1^H NMR (d_6_-acetone): δ 9.02 (1H, br s, –OH), 8.76 (1H, br s, –OH), 7.73 (1H, d, *J* = 15.8 Hz), 7.60 (1H, d, *J* = 15.8 Hz,), 7.72–7.52 (4H, m), 7.36 (1H, t, *J* = 8.0 Hz), 7.09 (1H, ddd, *J* = 8.8 Hz, 2.4 Hz, 1.0 Hz), 6.91 (2H, d, *J* = 8.8 Hz); ^13^C-NMR (d_6_-acetone): δ 189.7, 160.8, 158.5, 145.0, 140.9, 131.5, 130.5, 127.6, 120.5, 119.8, 116.7, 115.5, 113.8.

(*E*)*-1-(3-hydroxyphenyl)-3-(3-hydroxyphenyl)prop-2-en-1-one* (**1g**) [25]. ^1^H NMR(d_6_-acetone): δ 8.90 (1H, br s, –OH), 8.77 (1H, br s, –OH), 7.77 (2H, s, A_2_), 7.57 (1H, d, *J* = 7.6 Hz), 7.53 (1H, t, *J* = 2.2 Hz), 7.37 (1H, t, *J* = 7.8 Hz), 7.31–7.22 (3H, m), 7.11 (1H, ddd, *J* = 7.6 Hz, 2.6 Hz, 1.0 Hz), 6.96–6.90 (1H, m). ^13^C-NMR (d_6_-acetone): δ 189.9, 158.7, 158.6, 144.8, 140.5, 137.3, 130.8, 130.6, 122.9, 120.9, 120.8, 120.6, 118.5, 115.8, 115.6.

(*E*)-*1-(4-fluorophenyl)-3-(4-isopropoxyphenyl)prop-2-en-1-one* (**1h**). ^1^H-NMR (CDCl_3_, 300 MHz): δ 8.07–8.02 (2H, m), 7.76 (1H, d, *J* = 15.6 Hz), 7.35 (1H, d, *J* = 15.6 Hz), 7.23–7.14 (4H, m), 6.91 (2H, d, *J* = 8.4 Hz), 4.63 (1H, septa, *J* = 6.3 Hz), 1.41 (6H, d, *J* = 6.3 Hz) ; EI-MS *m*/*z* (relative intensity%): 284 (M^+^, 49), 241 (100), 225 (11), 147 (10), 123 (9); HRMS Calcd for C_18_H_17_FO_2_: 284.1213, Found: 284.1213.

(*E*)-*3-(4-isopropoxyphenyl)-1-phenylprop-2-en-1-one* (**1i**).^1^H-NMR (CDCl_3_): δ 8.01 (2H, dd, *J* = 8.1 Hz, 1.5 Hz), 7.79 (1H, d, *J* = 15.6 Hz), 7.59–7.53 (3H, m), 7.50 (2H, t, *J* = 8.4 Hz), 7.42 (1H, d, *J* = 15.6 Hz), 6.94 (2H, d, *J* = 8.4 Hz), 4.69 (1H, septa, *J* = 6.3 Hz), 1.43 (6H, d, *J* = 6.3 Hz); EI-MS *m*/*z* (relative intensity%): 266 (M^+^, 64), 223 (100), 147 (16); HRMS Calcd for C_18_H_18_O_2_: 266.1307, Found: 266.1315.

(*E*)-*3-(4-Fluoro-phenyl)-1-(4-isopropoxy-3-methoxy-phenyl)-propenone* (**1j**). ^1^H-NMR(CDCl_3_): δ 7.77(1H, d, *J* = 15.6 Hz), 7.67–7.61(3H, m), 7.49 (1H, d, *J* = 15.6 Hz), 7.14–7.08 (2H, td, *J* = 8.7, 2.1 Hz), 6.93 (1H, d, *J* = 8.4 Hz), 4.69 (1H, septa, *J* = 6.0 Hz), 3.95 (3H, s), 1.43 (6H, d, *J* = 6.0 Hz). ^13^C-NMR(CDCl_3_): δ 188.2, 165.4, 162.1, 151.8, 150.0, 142.4, 131.3, 130.2, 130.1, 122.8, 121.3, 116.1, 115.8, 112.6, 111.3, 71.1, 56.0, 21.8; EI-MS *m*/*z* (relative intensity%): 314(M^+^, 70), 272(98), 271(100), 241(55), 124(87), 123(82), 95(75); HRMS Calcd for C_18_H_17_FO_2_:314.1318 Found: 314.1315.

(*E*)-*3-(4-isopropoxy-3-methoxyphenyl)-1-phenylprop-2-en-1-one* (**1k**).^1^H-NMR(CDCl_3_): δ 8.01 (2H, dd, *J* = 8.1 Hz, 1.5 Hz), 7.76 (1H, d, *J* = 15.6 Hz), 7.58 (1H, td, *J* = 7.2 Hz, 1.5 Hz), 7.50 (2H, td, *J* = 7.5 Hz, 1.5 Hz), 7.38 (1H, d, *J* = 15.6 Hz), 7.21(1H, dd, *J* = 8.4 Hz, 2.1 Hz), 7.17 (1H, d, *J* = 2.1 Hz), 6.91(1H, d, *J* = 8.4 Hz), 4.62 (1H, septa, *J* = 6.0 Hz), 3.92 (3H, s), 1.41 (6H, d, *J* = 6.0 Hz); ^13^C-NMR (CDCl_3_): δ 190.2, 150.1, 149.8, 144.8, 138.3, 132.3, 128.3, 128.2, 127.6, 122.8, 119.7, 114.4, 110.9, 71.0, 55.8, 21.8; HRMS Calcd for C_19_H_20_O_3_: 296.1412, Found: 296.1410.

(*E*)-*3-(4-isopropoxy-3-methoxyphenyl)-1-p-tolylprop-2-en-1-one* (**1l**).^1^H-NMR(CDCl_3_): δ 7.93 (2H, d, *J* = 8.1 Hz), 7.75 (1H, d, *J* = 15.6 Hz), 7.38 (1H, d, *J* = 15.6 Hz), 7.30 (2H, d, *J* = 8.1 Hz), 7.20 (1H, dd, *J* = 8.4, 1.8 Hz), 7.16 (1H, d, *J* = 1.8 Hz), 6.91(1H, d, *J* = 8.4 Hz), 4.62 (1H, septa, *J* = 6.0 Hz), 3.92 (3H, s), 2.44 (3H, s), 1.41 (6H, d, *J* = 6.0 Hz); ^13^C-NMR (CDCl_3_): δ 189.9, 150.2, 149.8, 144.5, 143.2, 135.8, 129.1, 128.5, 127.8, 122.8, 119.8, 114.4, 110.9, 71.2, 55.9, 21.9, 21.5; EI-MS *m*/*z* (relative intensity%): 310(M^+^, 100), 268(96), 253(98), 145(53), 119(92), 91(93); HRMS Calcd for C_20_H_22_O_3_:310.1569, Found: 310.1563.

(*E*)-*1-(3,4-diisopropoxyphenyl)-3-(4-isopropoxy-3-methoxyphenyl)prop-2-en-1-one* (**1m**). ^1^H-NMR (CDCl_3_): δ 7.74 (1H, d, *J* = 15.6 Hz), 7.65 (1H, d, *J* = 8.4 Hz), 7.64 (1H, s), 7.38 (1H, d, *J* = 15.6 Hz), 7.20 (1H, d, *J* = 8.4 Hz), 7.16 (1H, s), 6.95 (1H, d, *J* = 8.0 Hz), 6.90 (1H, d, *J* = 8.4 Hz), 4.62 (2H, septa, *J* = 5.6 Hz), 4.45 (1H, septa, *J* = 6.4 Hz), 3.92 (3H, s), 1.40 (3H, d, *J* = 5.6 Hz), 1.39 (3H, d, *J* = 6.0 Hz), 1.36 (3H, d, *J* = 6.0 Hz); ^13^C-NMR(75 MHz): δ 188.9, 153.6, 150.2, 149.7, 148.5, 144.1, 131.6, 128.0, 123.4, 122.7, 119.7, 118.1, 115.0, 114.5, 110.9, 72.6, 71.7, 71.3, 56.1, 22.2, 22.1, 22.0; HRMS Calcd for C_25_H_32_O_5_: 412.2250, Found: 412.2252.

(*E*)-*3-(4-fluorophenyl)-1-phenylprop-2-en-1-one* (**1n**) [55]. ^1^H-NMR (CDCl_3_): δ 8.02 (2H, d, *J* = 7.3 Hz), 7.77 (1H, d, *J* = 15.6 Hz), 7.66–7.56 (3H, m), 7.49–7.42 (3H, m), 7.10 (2H, t, *J* = 8.7 Hz).

(*E*)*-4-(3-Oxo-3-phenylprop-1-en-1-yl)benzonitrile* (**1o**) [55]. ^1^H-NMR (CDCl_3_): δ 8.01 (2H, d, *J* = 7.4 Hz), 7.78–7.70 (3H, m), 7.68 (2H, d, *J* = 8.6 Hz), 7.64–7.57 (2H, m), 7.51 (2H, t, *J* = 8.6 Hz).

(*E*)-*1-Phenyl-3-(p-tolyl)prop-2-en-1-one* (**1p**) [56]. ^1^H-NMR (CDCl_3_): δ 8.07–7.99 (2H, m), 7.80 (1H, d, *J* = 15.6 Hz), 7.61–7.46 (6H, m), 7.24 (2H, t, *J* = 7.6 Hz), 2.40 (3H, s); ^13^C-NMR (CDCl_3_): δ 190.6, 144.9, 141.0, 138.3, 132.6, 132.1, 129.7, 128.5, 128.4, 128.4, 121.0, 21.49.

(*E*)-*1,3-Bis(4-nitrophenyl)prop-2-en-1-one* (**1q**) [57]. ^1^H-NMR (CDCl_3_): δ 7.97–7.98 (2H, m), 7.53–7.52 (2H, m), 8.01–8.03 (2H, m), 8.17 (2H, m), 6.88 (1H, d, *J* = 15.6 Hz), 6.37 (1H, d, *J* = 15.6 Hz).

(*E*)-*1-(4-Nitrophenyl)-3-phenylprop-2-en-1-one* (**1r**) [58]. ^1^H-NMR (CDCl_3_): δ 8.34 (2H, d, *J* = 8.4 Hz), 8.15 (2H, d, *J* = 8.4 Hz), 7.85 (1H, d, *J* = 15.6 Hz), 7.69–7.63 (2H, m), 7.49 (1H, d, *J* = 15.6 Hz), 7.47–7.43 (3H, m); ^13^C-NMR (CDCl_3_): δ 189.0, 150.0, 146.8, 143.0, 134.2, 131.2, 129.4, 129.1, 128.7, 123.8, 121.2.

(*E*)-*1-(4-Methoxyphenyl)-3-phenylprop-2-en-1-one* (**1s**) [55]. ^1^H-NMR (CDCl_3_): δ 8.03 (2H, d, *J* = 8.8 Hz), 7.80 (1H, d, *J* = 15.6 Hz), 7.66–7.63 (2H, m), 7.56 (1H, d, *J* = 15.6 Hz), 7.44–7.40 (3H, m), 6.95 (2H, d, *J* = 8.8 Hz), 3.86 (3H, s).

(*E*)-*1-(4-Fluorophenyl)-3-phenylprop-2-en-1-one* (**1t**) [56]. ^1^H-NMR (CDCl_3_): δ 8.06 (2H, dd, *J* = 8.7, 5.5 Hz), 7.82 (1H, d, *J* = 15.6 Hz), 7.63–7.61 (2H, m), 7.51 (1H, d, *J* = 15.6 Hz), 7.46–7.37 (3H, m), 7.17–7.13 (2H, m); ^13^C-NMR (CDCl_3_): δ 188.7, 165.5 (d, *J*_C–F_ = 252.9 Hz), 144.9, 134.7, 134.4 (d, *J*_C–F_ = 2.9 Hz), 131.0 (d, *J*_C–F_ = 9.2 Hz), 130.5, 128.9, 128.4, 121.5, 115.7 (d, *J*_C–F_ = 21.7).

### 4.2. Cell Culture 

Human monocytic THP-1 cell line was originally obtained from the Bioresource Collection and Research Center (BCRC, Taiwan, catalog #60430). The cell line was cultured in RPMI 1640 medium (Corning, Corning, NY, USA, catalog #10-040-CMR) with 10% fetal bovine serum (FBS) (catalog #10437-028), MEM Non-Essential Amino Acids Solution (NEAA) (gibco, Grand Island, NY, USA, catalog #11140-050), sodium pyruvate (gibco, catalog #11360-070), penicillin (100 U/mL), and streptomycin (100 µg/mL) in a humidified incubator with 5% CO_2_ at 37 °C. For each experiment, equal amount (1 × 10^6^/mL) of THP-1 cells were incubated with PMA (100 ng/mL) for 3 days to differentiate into macrophages adherent to dishes. For each analysis, cells were incubated in starvation medium containing 0.1% FBS, MEM NEAA, penicillin, and streptomycin.

### 4.3. Cell Viability Assay

The possible cytotoxic effect induced by chalcone compounds was measured by 3-[4,5-Dimethylthiazol-2-yl]-2,5-diphenyl tetrazolium bromide (MTT) assay. THP-1 monocytes were seeded into 96-well plates containing 100 μL of culture medium with PMA to differentiate into macrophages. After incubating THP-1 macrophages for 3 days, they were treated with a series of chalcone compounds (10 μM) in 100 μL of starvation medium for 24 h. After treatment with target compounds, 25 μM of MTT (5 mg/mL) was added to each well; the cells were incubated at 37 °C for 6 h. MTT was then replaced with 100 μL of dimethyl sulfoxide, and the amount of dissolved reduced MTT crystals was measured using an enzyme-linked immunosorbent assay reader.

### 4.4. Flow Cytometry

THP-1 cells were treated with PMA in 12-well culture plates (5 × 10^5^ cells/well) for 3 days to differentiate, and subsequently, THP-1 macrophages were treated with various chalcone compounds for 24 h. The harvested cells were digested by 0.5 M EDTA and washed with phosphate-buffered saline. After washing, cells were fixed with 0.5% paraformaldehyde/PBS and stained with ABCA1 antibody (abcam, catalog #ab18180, 1:250) for 1 h at 4 °C. The cells were then stained using the second antibody with fluorescein isothiocyanate (FITC)-conjugated anti-mouse IgG for 40 min in a dark place at 4 °C. The expression of ABCA1 was analyzed using FACSCalibur (BD Biosciences, Franklin Lakes, NJ, USA). 

### 4.5. Western Blot

At first, the cells were lysed using RIPA lysis buffer (Millipore, Burlington, MA, USA, catalog #20-188) and protease and phosphatase inhibitor (Thermo, Waltham, MA, USA, catalog #78442). Whole cell lysates were harvested through centrifugation and collection of the supernatants. After calculating the protein concentration using Bradford reagent (Bio-Rad, Hercules, CA, USA) with BSA as a standard, equal amounts of protein sample were added to 5X loading buffer (iNtTRON Biotechnology, catalog #IBS-BS002). The protein samples were resolved on 8–12% SDS-PAGE gels and transferred onto membranes using a wet transfer system. Membranes were blocked in 5% (*wt*/*vol*) BSA (Affymetrix, Santa Clara, CA, USA, catalog #9048-46-8) in TBS-T (50 mM Tris/HCL, pH 7.6, 150 mM NaCl and 0.1% (*vol*/*vol*) Tween-20) for 1 h at room temperature. Subsequently, the membranes were incubated with primary antibodies that included those for ABCA1 (abcam, catalog #ab18180, 1:5000), LXRα (Santa Cruz Biotechnology, Inc., Santa Cruz, CA, USA, catalog #sc-1202, 1:1000), PPARγ (Santa Cruz, catalog #sc-7196, 1:1000) and Actin (Thermo, catalog #MA5-15739, 1:10000) diluted in 2.5% (*wt*/*vol*) BSA in TBS-T and then with the appropriate horseradish peroxidase (HRP)-conjugated secondary antibodies diluted in 2.5% (*wt*/*vol*) BSA in TBS-T. Immunodetection of relative intensities was performed using ImageQuant LAS 4000 and quantified using ImageJ.

### 4.6. Quantification of mRNA 

All RNA samples were isolated from a 6-cm-dish of fresh human THP-1 macrophage (2 × 10^6^ cells per dish) using RNeasy kit (QIAGEN, Hilden, Germany, catalog #74106) according to manufacturer instructions. The concentration and purity were evaluated by Nanodrop spectrophotometer. 1000 ng RNA were reverse transcripted into cDNA using an iScript cDNA synthesis kit (BIO-RAD, catalog #170-8891) following the manufacturer protocol. KAPA SYBR FAST kit (catalog #kk4609) were used in qRT-PCR and 20 ng cDNA with 200 nM primers were applied in each reaction. qRT-PCR reaction was carried out by Roche LC480 2X. The cycling program consists of an initial activation at 95 °C for 3 min, followed by 40 cycles of 95 °C for 10 s, 60 °C for 20 s and 72 °C for 1 s. Immediately after amplification, melt-curve analysis was performed from 65 °C to 97 °C. The quantification of mRNA was performed following MIQE guidelines [59]. The calibration curve for a primer set was generated from a series of dilution of the cDNA template. Each primer efficiency was then calculated (E = 10^−1/slope^). The relative quantification of target mRNA was calculated based on the equation $\left(RQ=\frac{E_{target}{}^{\Delta \mathrm{Ct}\left(target\right)}}{E_{reference}{}^{\Delta \mathrm{Ct}\left(reference\right)}}\right)$ and GAPDH was served as the reference gene. For all genes determined in our study, single peak was shown in melting curve which indicated the PCR amplification was specific. There was no Cq value of NTC (no template control) detected in all primer sets. The detailed information regarding primer sequences, efficiencies, and calibration curves was provided in the supplementary information (Supplementary Table S1, Figure S1). 

### 4.7. Quantification of MicroRNA 

All miRNA samples were isolated from a 6-cm-dish of human THP-1 macrophage (2 × 10^6^ cells per dish) using miRNeasy kit (QIAGEN, catalog #1038703) following manufacturer instructions. The concentration and purity were evaluated by Nanodrop spectrophotometer. cDNA was then synthesized from 1000 ng miRNA using miScript II RT kit with HiFlex Buffer (QIAGEN, catalog #218161). Each cDNA synthesis was then diluted by RNase-free water (20 ng cDNA in each well) and mixed with miScript SYBR Green PCR kit (QIAGEN, catalog #218073). qRT-PCR was optimized by Roche LC480 2X. The three-step cycling program was settled as an initial activation at 95 °C for 15 min, followed by 40 cycles of 95 °C for 15 s, 55 °C for 30 s and 70 °C for 30 s. The calibration curve for a primer set was generated from a series of dilution of the cDNA template. Each primer efficiency was then calculated to adjust corresponding PCR results. The expression of miRNA was analyzed by relative quantification with comparative CT method and normalized to RNU6-2 as the universal invariant reference primer (∆Cq), followed by a comparison of vehicle sample (∆∆Cq). The detailed primer information was provided in the supplementary information. (Supplementary Table S2, Figure S1).

### 4.8. Statistical Analysis

Data were expressed as mean ± SEM for at least three independent experiments. Statistical data were analyzed using a Student’s unpaired *t* test or one-way ANOVA; *p* < 0.05 was considered statistically significant. 

## 5. Conclusions

Privileged structures and chemical properties make certain chalcone derivatives potential for modifying new drug designs and developments for treating atherosclerotic diseases associated with lipid dysmetabolism. With their unique and multiplex properties, certain chalcone derivatives are promising antiatherosclerotic agents that warrant further investigation.

## Supplementary Materials

The following are available online: Table S1: The sequences and efficiencies of mRNAs for real-time quantitative PCR assay; Table S2: The sequences and efficiencies of miRNAs for real-time quantitative PCR assay; Figure S1: Standard curves of the significant primers.
