# Supplementary material for: Chalcone Derivatives Enhance ATP-Binding Cassette Transporters A1 in Human THP-1 Macrophages
Source: Molecules. 2018 Jul 3;23(7):1620. doi: 10.3390/molecules23071620 (PMC6100038; doi:10.3390/molecules23071620)
Supplement: Supplementary file 1 [file molecules-23-01620-s001.pdf]

Supplementary Materials

Article

Chalcone Derivatives Enhance ATP-Binding Cassette Transporters A1 in Human THP-1 Macrophages

I-Jou Teng [1,†], Min-Chien Tsai [2,†], Shao-Fu Shih [3], Bi-Feng Tsuei [4], Hsin Chang [5], Yi-Ping Chuang [6], Chin-Sheng Lin [3], Ching-Yuh Chern [5,#] and Sy-Jou Chen [7,#,*]

1 Graduate Institute of Medical Sciences, National Defense Medical Center, Taipei, 11490, Taiwan (R.O.C); topy900703@gmail.com
2 Department of Physiology and Biophysics, Graduate Institute of Physiology, National Defense Medical Center, Taipei, 11490, Taiwan (R.O.C); mctsaisyy@gmail.com
3 Division of Cardiology, Department of Medicine, Tri-Service General Hospital, National Defense Medical Center, Taipei, 11490, Taiwan (R.O.C); evarei080@gmail.com (S.-F.S.); littlelincs@gmail.com (C.-S.L.)
4 Graduate Institute of Life Sciences, National Defense Medical Center, Taipei, 11490, Taiwan (R.O.C); befun1214@gmail.com
5 Department of Applied Chemistry, National Chiayi University, Chiayi City, 60004, Taiwan (R.O.C); s1060317@mail.ncyu.edu.tw (H.C.); cychern@mail.ncyu.edu.tw (C.-Y.C.)
6 Department and Graduate Institute of Microbiology and Immunology, National Defense Medical Center, Taipei, 11490, Taiwan (R.O.C); ypchuang@mail.ndmctsgh.edu.tw
7 Department of Emergency Medicine, Tri-Service General Hospital, National Defense Medical Center, Taipei, 11490, Taiwan (R.O.C); syjou.chen@gmail.com
† I-Jou Teng and Min-Chien Tsai contribute equally to this work.
# Ching-Yuh Chern and Sy-Jou Chen contribute equally to this work.
* Correspondence: syjou.chen@gmail.com; Tel: 886-2-8792-3311

Received: 22 May 2018; Accepted: 29 June 2018; Published: 3 July 2018

Table S1. The sequences and efficiencies of mRNAs for real-time quantitative PCR assay.

	Gene symbol	Sequence ID	Amplicon length	Sequence	Efficiency
**GAPDH**	Homo sapiens glyceraldehyde-3-phosphate dehydrogenase (GAPDH), transcript variant 6, 3, 1, 7, 4	NR_152150.1<br>NM_001289745.2<br>NM_002046.6<br>NM_001357943.1<br>NM_001289746.1	767<br>1401<br>1309<br>1235<br>1407	5'-ATGGGGAAGGTGAAGGTCG-3' (forward)<br>5'-TAAAAGCAGCCCTGGTGACC-3'(reverse)	1.964
**ABCA1**	Homo sapiens ATP binding cassette subfamily A member 1 (ABCA1), mRNA	NM_005502.3	10515	5'-GGTGATGTTTCTGACCAATGTGA-3′(forward)<br>5'-TGTCCTCATACCAGTTGAGAGAC-3'(reverse)	1.776
**ABCG1**	Homo sapiens ATP binding cassette subfamily G member 1 (ABCG1), transcript variant 2-7, mRNA	NM_207629.1<br>NM_207628.1<br>NM_207627.1<br>NM_207174.1<br>NM_004915.3<br>NM_016818.2	2946<br>3060<br>3142<br>2983<br>3018<br>2982	5'-AGGGATTTGGGTCTGAACTG-3′(forward)<br>5'-GGTCTCTCTTGTGGTCTGAG-3′(reverse)	1.575
**LXR-α**	Homo sapiens nuclear receptor subfamily 1 group H member 3 (NR1H3), transcript variant 1-5, mRNA	NM_005693.3<br>NM_001130101.2<br>NM_001130102.2<br>NM_001251934.1<br>NM_001251935.1	1939<br>1759<br>1748<br>1887<br>1928	5′-AAGCCCTGCATGCCTACGT-3′(forward)<br>5′-TGCAGACGCAGTGCAAACA-3′ (reverse)	2.182
**CCL2**	Homo sapiens C-C motif chemokine ligand 2 (CCL2), mRNA	NM_002982.3	730	5'-GATCTCAGTGCAGAGGCTCG-3′(forward)<br>5'-TGCTTGTCCAGGTGGTCCAT-3′(reverse)	1.919

Table S2. The sequences and efficiencies of miRNAs for real-time quantitative PCR assay.

	Catalog No.	miRBase ID	miRBase Accession	Mature miRNA sequence	Efficiency
**Hs_miR-155*_1**	MS00008778	hsa-miR-155-3p	MIMAT0004658	CUCCUACAUAUUAGCAUUAACA	2.0157
**Hs_miR-758_2**	MS00010563	hsa-miR-758-3p	MIMAT0003879	UUUGUGACCUGGUCCACUAACC	1.8573
**Hs_miR-10b*_1**	MS00008421	hsa-miR-10b-3p	MIMAT0004556	ACAGAUUCGAUUCUAGGGGAAU	2.0916
**Hs_miR-145*_1**	MS00008708	hsa-miR-145-3p	MIMAT0004601	GGAUUCCUGGAAAUACUGUUCU	1.9844
**Hs_miR-33a*_1**	MS00009492	hsa-miR-33a-3p	MIMAT0004506	CAAUGUUUCCACAGUGCAUCAC	2.0742
**Hs_miR-106b*_1**	MS00008400	hsa-miR-106b-3p	MIMAT0004672	CCGCACUGUGGGUACUUGCUGC	2.0461
**Hs_miR-144_4**	MS00020328	hsa-miR-144-3p	MIMAT0000436	UACAGUAUAGAUGAUGUACU	2.3318
**Hs_miR-206_1**	MS00003787	hsa-miR-206	MIMAT0000462	UGGAAUGUAAGGAAGUGUGUGG	1.9000
**Hs_RNU6-2_11**	MS00033740	Transcript(s) NR_002752 (107 bp)			2.0303

Figure S1. Standard curves of the significant primers.

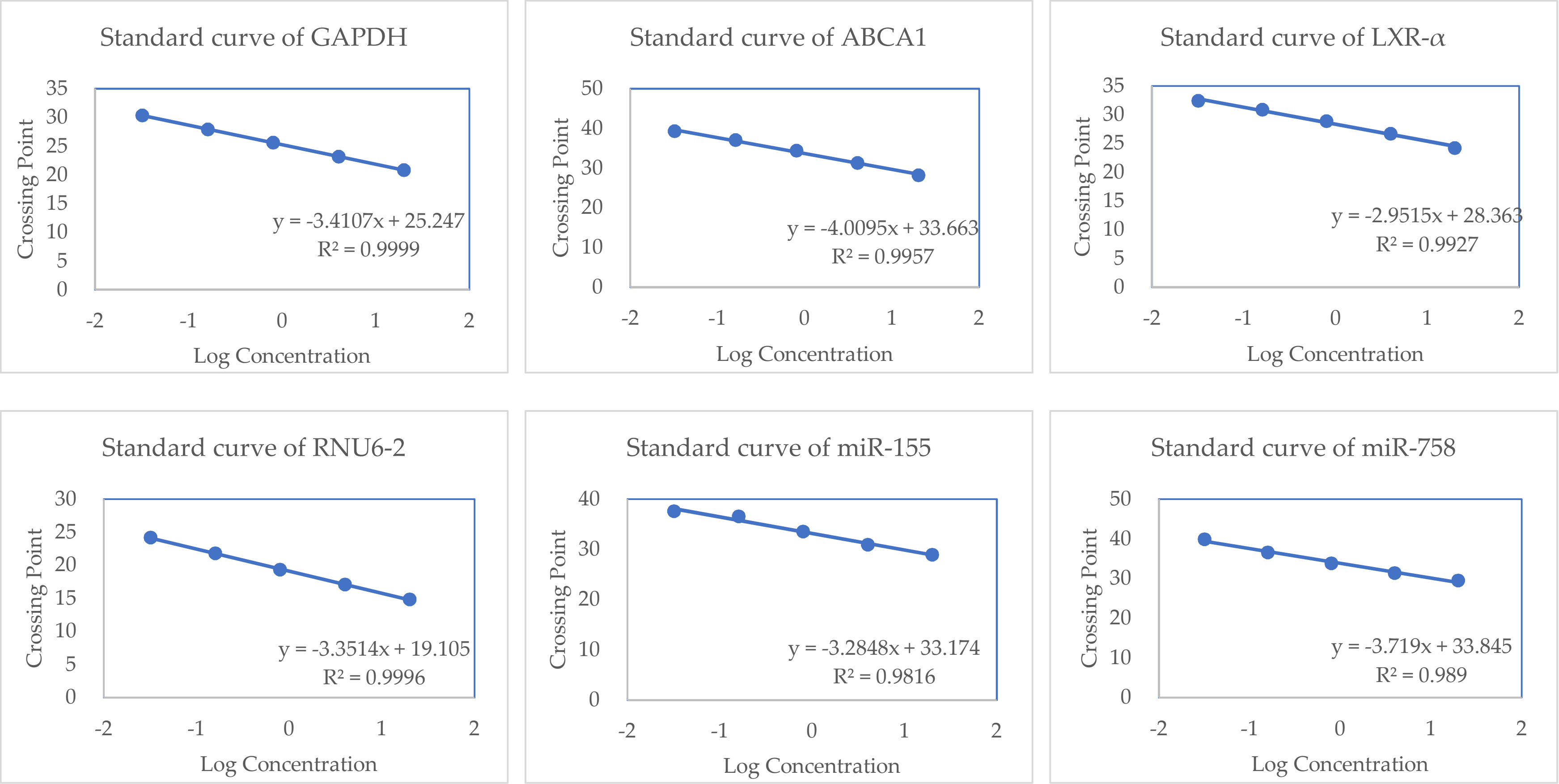

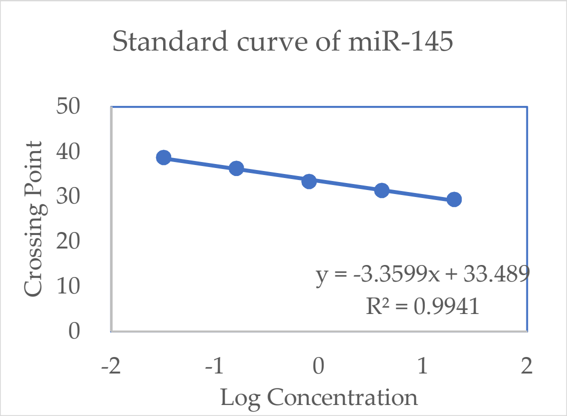
Standard curve of miR-145
Crossing Point
Log Concentration
50
40
30
20
10
0
-2
-1
0
1
2
y = -3.3599x + 33.489
R² = 0.9941

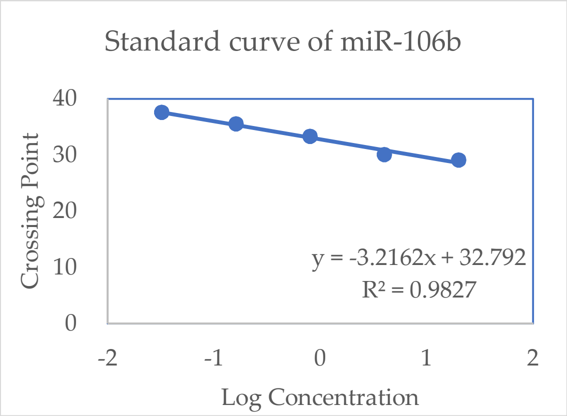
Standard curve of miR-106b
Crossing Point
Log Concentration
40
30
20
10
0
-2
-1
0
1
2
y = -3.2162x + 32.792
R² = 0.9827

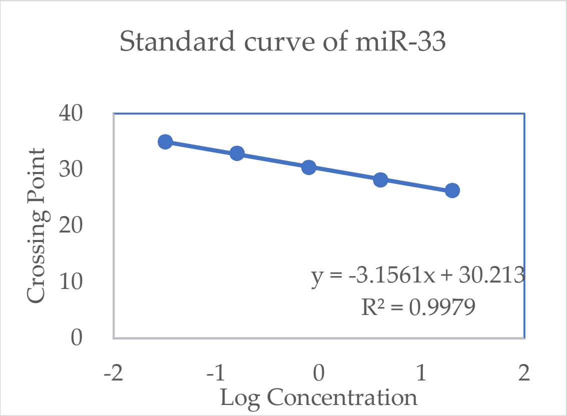
Standard curve of miR-33
Crossing Point
Log Concentration
40
30
20
10
0
-2
-1
0
1
2
y = -3.1561x + 30.213
R² = 0.9979

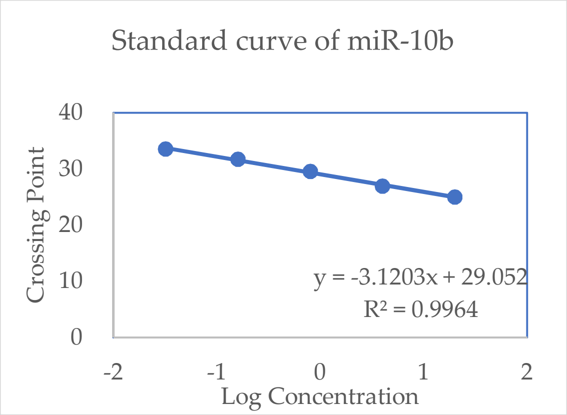
Standard curve of miR-10b
Crossing Point
Log Concentration
40
30
20
10
0
-2
-1
0
1
2
y = -3.1203x + 29.052
R² = 0.9964
